# Impact of Processing Method on AQF Functionality in Bakery Items

**DOI:** 10.3390/foods12112210

**Published:** 2023-05-31

**Authors:** Dana Edleman, Clifford Hall

**Affiliations:** 1 Department of Food Science and Nutrition, University of Minnesota, St. Paul, MN 55108, USA; edlem002@umn.edu; 2 Department of Dairy and Food Science, South Dakota State University, Brookings, SD 57007, USA

**Keywords:** AQF, cake, cookie, chickpea, egg, reverse osmosis, drying

## Abstract

Aquafaba (AQF) has the unique ability to foam like egg whites and is a waste product of cooked chickpea that is not currently utilized by the food industry. Thus, the goal of this research was to concentrate the solids by reverse osmosis (cAQF) followed by drying. Dried AQF was prepared by cooking chickpea in excess water. After removal of the chickpea, the liquid AQF was subjected to reverse osmosis followed by freeze, tray, or spray drying. The resulting AQF products were incorporated into standard cake mix and sugar cookie formulas. Hardness, gumminess, and chewiness of cakes made with eggs were significantly higher compared to the cakes made with AQF. Spread factor was significantly greater for cookies made with AQF compared to eggs while hardness was significantly lower in cookies with AQF. Higher flavor and overall acceptability scores were observed in cookies made with AQF compared to cookies made with egg. However, sensory characteristics were generally not different among cakes. In general, cAQF and spray-dried AQF tended to produce cakes and cookies with the best quality and sensory characteristic. This research supports the use of RO and drying methods in producing AQF ingredients for baking applications.

## 1. Introduction

Chickpeas are a source of protein that can be used in a variety of food applications. Hummus and canned products are two of the most common uses of whole chickpeas. Aquafaba (AQF) is the liquid remaining after cooking chickpeas and is currently a waste product in hummus production and canning. Furthermore, the liquid in a canned chickpea is also a source of AQF. The discovery of AQF and its applications as a vegan egg substitute sprouted on the internet in 2015, making it a relatively new and minimally studied food ingredient. However, the interest of chefs and home cooks for vegan egg white substitutes in baking and confectionary applications has been a driver in the development of culinary food items with AQF [1]. Furthermore, egg replacement in baked goods may be desired by bakeries and consumers due to allergen labeling requirements and increased ingredient costs [2]. Eggs have both emulsification and foaming properties that makes them functional in many food applications. The functionality is attributed to a number of components but lipoproteins in the yolk contribute emulsification while globulins and glycoproteins contribute the foaming and foam stabilizing properties of egg white, respectively [3,4,5]. Thus, the desire to replace eggs in food applications with AQF means that a significant body of knowledge is still needed before AQF can be considered as an egg replacer. The scientific community has just recently started to better understand AQF composition, functionality, and factors that impact AQF recovery.

Although chickpea seed has been the primary pulse in many AQF studies [6,7], other pulse seeds such as peas, edible beans, faba beans, lima beans, lentils, soybeans, and black soybeans can be a source of AQF [8,9,10,11,12]. Variability in composition and properties of the AQF depend on factors such as cultivar [13] and processing method to obtain AQF [14,15,16]. As a result, one cannot expect to obtain the same results, and thus comparisons between studies should be made with caution. Regardless of the differences in results obtained by researchers, AQF is an effective emulsifying and foaming agent.

The CDC leader cultivar of chickpea had the lowest AQF yield but the best emulsion activity and stability [13]. These authors indicated that the dry matter content of AQF likely contributed to the emulsion properties since the original composition of chickpea used in the making of AQF did not correlate to emulsion properties. Furthermore, the protein concentration of the AQF itself did not correlate well with emulsion properties [17]. This suggests that multiple compounds are likely responsible for emulsification properties. However, saponins were theorized to be responsible for the foaming activity Shim et al. [6] assessed AQF obtained from canned chickpeas and found twenty different compounds in the AQF including sugars, alanine, choline, and alcohols. However, no correlation between AQF functionality and composition has been definitively established. Regardless, AQF has the unique ability to emulsify and foam, resulting in a foam comparable to whipped egg whites. The emulsification properties of AQF have been demonstrated in mayonnaise [18,19,20,21]. However, sensory evaluation of the mayonnaise was not reported and was suggested to be an important evaluation to establish consumer acceptance [22].

The foaming properties of AQF have been evaluated in confectionary and bakery products. In confectionery meringues, the AQF source [8] and acidifications [23] impacted quality. For example, AQF obtained from yellow pea produced meringues with the best sensory properties while the green lentil AQF received the lowest sensory scores. However, the foamability was not significantly different between the peas and lentils, but these seeds produced significantly more foam than haricot bean and chickpea AQFs. Unlike emulsification, foaming properties tended to be correlated to protein content [8]. Tufaro and Cappa [23] observed that AQF in the presence of lactic acid produced foams with the greatest overrun and the least amount of syneresis and shrinkage. A cupcake containing lentil AQF had lower hardness, chewiness, gumminess, and cohesiveness than cupcakes made with eggs [9]. Similarly, sponge cake made with AQF tended to have lower chewiness, springiness, and cohesiveness compared to sponge cakes made with eggs [7]. During cake baking, the retention of air cells is important for the cake structure and textural properties. The cake structure is heavily dependent on the disulfide bond formation between egg proteins or between egg protein and wheat protein during the baking process [24,25]. Furthermore, starch gelatinization contributes structure through interactions with protein to form cell wall structure for air cells within the cake [26]. Cakes tend to collapse or have less springiness if insufficient cell wall structure is developed during baking. Thus, differences observed in cakes made with AQF compared to eggs likely lacked sufficient cell wall structure. In contrast, cakes made with varying AQF and fat levels tended to produce cakes with slightly higher specific volumes than cakes made with palm oil without AQF [27]. However, no differences in sensory scores between cakes made with and without AQF were observed. In general, the addition of AQF to gluten-free bread formulation resulted in breads with high specific volumes and softer crumb texture but more bitterness, compared to other gluten free breads [28].

The literature supports AQF for its unique functionality. However, it is not widely used by the food industry due to the excessive cost of transporting water, which accounts for approximately 91–95% of AQF composition [1]. To address this limitation and utilize this waste, methods to remove water and maintain functionality are needed to make this a feasible ingredient. Limited studies have been reported on the use of dry AQF [1,29].

Concentration and drying would allow for the waste product to be converted into a usable ingredient. The purpose of this project was to create concentrated liquid AQF using reverse osmosis followed by drying. The specific objectives were to assess the functionality and application, in cakes and cookies, of concentrated and dried AQF obtained from the cooking water of chickpeas. In this research, only the Frontier cultivar was assessed for AQF production. The focus of this research was on processing approaches to produce dried AQF; thus, reducing the variables to only those associated with the processes would help to minimize the confounding impacts of other factors such as cultivar. The hypothesis of this research was that concentration and drying methods will impact the quality and functionality of chickpea AQF in model foam and emulsion systems or impact the quality of cakes and cookies. In this research, liquid AQF was concentrated by reverse osmosis (cAQF) followed by drying using tray-, freeze-, and spray-drying operations. The AQF samples were incorporated into cakes and cookies, and quality and sensory characteristics were evaluated.

## 2. Materials and Methods

### 2.1. Materials

Dried chickpeas (400 pounds, Frontier cultivar) were obtained and cleaned to remove physical contaminants. Chemicals and solvents were obtained from Fisher Scientific. Remaining supplies were obtained through local vendors.

### 2.2. Preparation of Aquafaba

#### 2.2.1. Extraction and Concentration of Aquafaba

Aquafaba was prepared by cooking 200 pounds of dried chickpea at 93 °C in excess water (5 parts water to 1 part chickpea) for 4 h (Davis Dairy Plant, South Dakota State University). The cooking process was completed twice where a total of 400 pounds of chickpeas were processed. After removal of the chickpea, the liquid AQF was subjected to reverse osmosis to concentrate the solid. A Reverse Osmosis Membrane ROPlus-8038AN-SX5A (Solecta Filtration, Oceanside, CA, USA) was used to concentrate the solids. Other parameters included an operating temperature of 48.9 °C, feed rate of 15.14 L/min, and base pressure of 3.13 MPa. Under these conditions, the concentrate/retentate and permeate flow rate averaged 6.0 L/min and 7.9 L/min, respectively. The concentration of total solids (Brix) achieved in the retentate was approximately 13%. One fourth of the retentate was stored in a −20 °C freezer until further evaluations were completed. The replicate processing and subsequent concentrated AQF (cAQF) samples were maintained through the drying steps and analyses.

#### 2.2.2. Drying of Aquafaba

Drying of the cAQF (13% solids) was completed using tray, freeze, and spray drying. The cAQF was subjected to tray drying at 57 °C for 18 h using the Weston 160 L Dehydrator (Model No. 28- 0501-W; Southern Pines, NC, USA). Prior to drying, samples were placed on stainless trays and frozen. Freeze drying was completed using a HarvestRight freeze drier (North Salt Lake, UT, USA) using a 20 h cycle that started at −20 °C and ended at 0 °C. A GEA Niro Pilot Dryer (Model ND306; Columbia, MD, USA) with an air flow of 0.44 MPa, inlet temperature of 200 °C, and outlet temperature of 125 °C was used for spray drying.

Dried samples were ground using a cyclone mill (UDY; Fort Collins, CO, USA) through a 0.5 mm screen to obtain a dried powder. All dried powders were stored in polyethylene bags at ambient temperature.

### 2.3. Composition and Functionality

#### 2.3.1. Aquafaba Composition

The following proximate tests were conducted: total starch [30], moisture [31], ash [32], lipid [33], protein [34], and total dietary fiber [35]. Sugar content was analyzed by Minnesota Valley Testing Labs (New Ulm, MN, USA) using high-performance anionic-exchange chromatography with pulsed amperometric detection. Additionally, color analysis using the Hunter scale L* a* b* values (Konica Minolta CR-410 chroma meter; Ramsey, NJ, USA) was evaluated. Due to the liquid nature of the reverse osmosis treatment (i.e., cAQF), it did not undergo analysis; however, it is predicted the composition would be similar to the freeze-dried results as neither underwent heat treatment after the reverse osmosis process. The cAQF was 87% water and 13% solids. Thus, the predicted composition of the cAQF was determined by converting the dry weight basis obtained from freeze-dried sample to an as is basis (i.e., wet basis) using the following equation:

As is component = % component dry weight basis/(1 − % water as decimal)(1)

where the component represents protein, starch, etc.

#### 2.3.2. Functionality

Functionality tests were performed on the dried AQF and the cAQF samples. Foaming capacity and stability were determined using a modified method of Stone et al. [36]. A 1.00% (*w*/*w*) AQF solution was prepared by mixing 0.5 g of dried AQF with 49.5 g of 10 mM sodium phosphate buffer (pH 7.00). To account for differences in moisture, the foaming of the cAQF was prepared using different amounts of material. The cAQF (3.85 g) was mixed with water (46.15 g) and sodium phosphate (0.082 g) for an equivalent solution of dry AQF (0.5 g) and 10 mM sodium phosphate buffer (49.5 g). The resulting solutions from the dry AQF and cAQF were stored overnight at 4 °C prior to homogenization. Afterward, 15 mL (Vli) of the AQF solution was transferred into a narrow 400 mL glass beaker and foamed using an Omni GLH 850 homogenizer (Omni International, Kennesaw, GA, USA) with a 20 mm probe at the speed of 8000 rpm for 5 min. Immediately following homogenization, the foam was transferred to a 100 mL graduated cylinder. Foam volume was recorded at time zero and after 30 min of storage at ambient conditions. Foaming capacity (FC) and foaming stability (FS) were determined using following equations, respectively,

%FC = (Vfi)/(Vli) × 100(2)

%FS = (Vft)/(Vfi) × 100(3)

where Vfi = volume of foam immediately after homogenization and Vft = volume of foam remaining after 30 min.

Emulsion activity (EA) and emulsion stability (ES) were determined on both the dried AQF and cAQF with a slight modification to a previous reported [37]. The dried AQF (1.25 g) was suspended in 48.75 g of 10 mM sodium phosphate buffer (pH 7.00) while the cAQF (9.5 g) was mixed with water (40.5 g) and sodium phosphate (0.082 g) for an equivalent solution prepared for the dry AQF. All solutions were stored overnight at 4 °C. The AQF solution (24.5 mL) was mixed with 24.5 mL of canola oil in a 200 mL beaker using an Omni Macro homogenizer at the speed of 8000 rpm for 3 min. For EA, 10 mL of the homogenized solution was transferred to 15 mL centrifuge tubes. The height of the entire emulsion was measured, followed by centrifugation at 1315× *g* for 5 min. The heights of the emulsified layer were noted after centrifugation. For ES, the remaining portion of the emulsion in the beaker was heated at 80 °C in a water bath for 30 min and then cooled to room temperature in a cold-water bath for 15 min. Ten mL of the obtained emulsion was then transferred into a 15 mL centrifuge tube. The height of the entire emulsion was taken followed by centrifugation at 1315× *g* for 5 min. The heights of emulsified layer were recorded. EA and ES were calculated using the following equations:

EA(%) = (Height of emulsified layer)/(Height of entire emulsion in tube) × 100(4)

ES(%) = (Height of emulsified layer)/(Height of entire emulsion in tube) × 100(5)

### 2.4. Application of Aquafaba in Foam, Cakes, and Cookies

#### 2.4.1. AQF Foam

Foaming evaluations were completed to assess the potential of the AQF to act as a meringue. The dried AQF were rehydrated at 21 °C to a total volume of 400 mL with distilled water at concentrations of 4%, 6%, 8%, and 13% (solids basis), while cAQF did not undergo drying but instead was diluted with water to create lower solid contents. This range of concentrations was selected based on preliminary findings that indicated no foaming benefits beyond the 13% AQF concentration while the original pre-concentrated AQF had a solids content around 4%. The viscosity of the solutions was measured using a viscometer (BYK Instruments, Fort Lauderdale, FL, USA), where measurements were corrected for density before measurements. The L1 spindle and 100 rpm operational speed were used during the viscosity determination, and the sample was poured into a narrow 200 mL beaker attached to the viscometer and filled to an appropriate fill notch on spindle.

Foams were created by whipping 100 g of AQF, i.e., fresh liquid or rehydrated, and cream of tartar (0.97 g) in a stand mixer (KitchenAid Artesian Stand Mixer; Benton Harbor, MI, USA) for 24 min. The sides of the bowl (4 L) were scraped with a spatula every four minutes. At which time, an increase in the mixer speed was adjusted during mixing using settings 1–8 on mixer. For example, the initial speed was set at setting 1 and after four minutes was increased to setting 2. Increasing the speed was conducted until setting 8 was reached at the last four minutes of mixing. The volume of the foam that resulted during mixing was transferred to a 1000 mL graduated cylinder. Foam capacity was determined as the volume of foam in the 1000 mL graduated cylinder using Equation (2). Foam stability was measured after 30 min by decanting the serum into a 100 mL graduated cylinder to measure foam separation. The direct measure of volume (mL) of serum was used to differentiate stability using the following equation:

%FS_m_ = (Vfi − Lft)/(Vfi) × 100 (6)

where Vfi = volume of foam immediately after mixing and Lft = volume (mL) of serum separated after 30 min.

#### 2.4.2. Cake Production

Round 8-inch yellow cakes were baked using the dried AQF and control cAQF to investigate the ability for the AQF to replace egg in a vanilla cake mix application (Jiffy Mix, Chelsea, MI, USA). Preliminary experiments showed a solution of AQF at a 6% concentration was the most effective at replacing the egg in a cake mix. Thus, the dried AQF was rehydrated in water (diluted in the case of the cAQF) to a 6% concentration based on solids content. This solution was included in the cake formulation (Table 1) at 152.7 g of 6% AQF and 1 box (255 g) cake mix. Batter was produced using a stand mixer fitted with the paddle attachment (KitchenAid Artesian Stand Mixer; Benton Harbor, MI, USA). The aquafaba was solubilized with water using a spatula in a 250 mL beaker and added to the bowl of a stand mixer with the cake mix. For the control cake, egg and water were mixed in a 250 mL beaker with a spatula and added to the bowl of a stand mixer with the cake mix. All cake batters were mixed on speed 4 for 3 min. Cakes were baked in a parchment lined 8-inch round pan at 177 °C (350 °F) for 23 min. Cakes were allowed to cool in the pan for 10 min before being removed from the pan and cooled on a rack. After 1 h, cakes were stored in containers covered with plastic wrap until evaluation on day 1. Cake height determination was performed using AACC approved method 10–91.01 [38] to compare cakes made with AQF and cakes made with eggs. A two-cycle compression test was conducted to determine cake texture according to a Modified AIBCAKE2/1 (TA.XT.Plus, Texture Technologies; Hamilton, MA, USA) method. For water activity and moisture analysis, cake samples were crumbled using a pestle and mortar. Water activity was measured using a water activity meter (Pre Aqualab, Decagon Devices, Pullman, WA, USA). Moisture content was determined by drying 2 g of sample in an oven (Isotemp, Fisher Scientific, Pittsburgh, PA, USA) for 2 h at 130 °C. For sensory evaluation, cakes were cut into 1-inch cross sections and then rotated 90° and cut into 1-inch sections for panelists to evaluate as described in Section 2.4.4.

#### 2.4.3. Cookie Production

Traditional sugar cookies were produced with the four AQF treatments used to replace the egg according to a modified method of Gohl [39]. The AQF cookies were compared to a control cookie made with egg (Table 2). Sugar and room temperature butter were creamed together in a stand mixer fitted with the paddle attachment (KitchenAid Artesian Stand Mixer; Benton Harbor, MI, USA) at speed 4 for 1.5 min. For the egg control cookies, egg was added followed by mixing at speed 4 for 30 s. Mixing was completed following the same operational parameters for the cAQF while the dried AQF also included the addition of water at this mixing step. The remaining dry ingredients were added, starting with mixing on speed 1 for 30 s to minimize dry ingredient loss and increasing to speed 4 for 90 s. The dough was rolled out onto a floured surface using a plastic rolling pin fitted with rubber bands to achieve a 6.35 mm thickness. A 57 mm diameter circular cutter was used to shape cookies. Cookies were placed onto a greased 31.7 × 43.9 × 2.5 cm cookie sheet and baked in an oven (Vulcan; Baltimore, MD, USA) at 177 °C for a total of 8 min, with a 180° pan rotation halfway through baking to ensure an even baking of cookies. Cookies were removed from pan and allowed to cool for 30 min before placing into plastic bags for storage. For water activity and moisture analysis, cookies were crumbled using a pestle and mortar. Water activity was measured using a water activity meter (Pre Aqualab, Decagon Devices, Pullman, WA, USA). Moisture content was determined by drying 2 g of sample in an aluminum tin in an oven (Isotemp, Fisher Scientific, Pittsburgh, PA, USA) for 2 h at 130 °C. For sensory evaluation (Section 2.4.4), smaller cookies were produced using the same method but with a 24 mm diameter cookie cutter, and baking time was reduced to a total of 6 min.

Cookie texture and shelf-life evaluation was conducted according to AACC approved method 10–54.01 [40]. The texture analyzer (TA.XT.Plus) performed a 3-point break test measuring the force needed to split the cookie with probe TA-92N and the bend rig set 2 inches apart. Additional settings included pre-test speed of 2.5 mm/s, test speed of 2.0 mm/s, post-test speed of 10 mm/s, a distance of 6 mm, trigger type of 20 g, automatic tare rate, and a data acquisition rate of 200 pps. Hardness and fracturability were evaluated on days 1, 4, 8, and 14.

#### 2.4.4. Sensory Evaluation

Sensory evaluation (protocol 2107008-EXM) was conducted for both cake and cookies, with sensory for each product type conducted independently, but in an identical manner. Panelists, obtained from students, faculty, and staff at South Dakota State University, evaluated a total of 5 samples of the following formulations: egg, tray-dried, spray-dried, freeze-dried, and cAQF as the treatments. Panelists were delivered samples in plastic cups with labeled 3-digit codes in a randomized order to avoid delivery order bias. The evaluation was conducted using a 9-point hedonic scale in increments from “Like Extremely” (score of 9) to “Dislike Extremely” (score of 1) for appearance, flavor, texture, and overall sensory characteristics for the product. The products were evaluated by 84 and 98 panelists for cookies and cakes, respectively.

#### 2.4.5. Statistical Methods

The processing of the AQF was conducted twice and composition analysis, viscosity, and water activity were conducted in duplicate measures (*n* = 4). Cake and cookies were prepared in duplicate batches based on processing replication. Cookie and cake physical measurements were completed in triplicate (*n* = 6). Emulsion and foaming properties were completed four times on the duplicate processing samples (*n* = 8). Data were analyzed using ANOVA (R Studio Version 1.4.1717) where significance was determined at *p* ≤ 0.05. Mean separation was completed using Tukey’s least significant difference (LSD).

## 3. Results and Discussion

### 3.1. Aquafaba Composition

**Figure 1:**
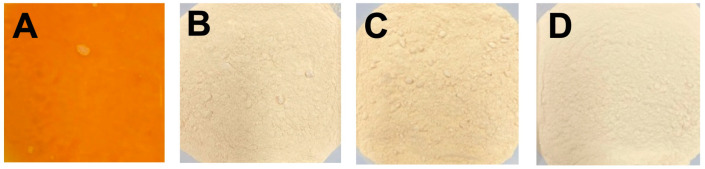
The visual appearance of aquafaba samples obtained by reverse osmosis ((**A**), cAQF) followed by freeze (**B**), tray (**C**), or spray (**D**) drying.

A total solid percentage of the aquafaba samples were measured at the cooking stage, after reverse osmosis, and after drying for both processing replicates. The concentration of total solids achieved during the cooking stage was 3.3%. After reverse osmosis, the total solids increased to approximately 13%. After drying, total solids reached 90.3%, 91.8%, and 95.0% for tray-, freeze-, and spray-dried samples, respectively. Differences in the appearance of the dry product were observed (Figure 1). Overall, the appearances of the dried samples were comparable to similarly dried AQF from chickpea [18]. For the dry products, the lightness observed in the spray- and freeze-dried AQF was supported by the L* values being higher, although not significant, than the L* values of the tray-dried sample (Table 3). The tray-dried sample had a yellow-brown appearance, which was supported by higher b* (yellowness) values. The values obtained in the spray- and freeze-dried methods followed the same trends as similarly processed AQF from pea and chickpea [10,18].

The composition of the AQF was predominantly protein, ash, sugar, and dietary fiber (Table 4 and Table 5). As expected, the drying processes substantially enhanced the nutrient composition compared to an estimated value for the cAQF sample. In general, the drying methods had little impact on the proximate composition except for sucrose. The tray-dried AQF had 1.5 percentage points lower sucrose (Table 5) concentration compared to the samples dried by spray- or freeze-drying methods. The tray-drying method required 18 h at 57 °C to dry the AQF, and during this time caramelization reaction may have contributed to the sucrose reduction. The increased browning (Figure 1) of the tray-dried samples is indirectly supported by the loss of sucrose during drying. In this study, the AQF was concentrated by a factor of approximately 3.25 compared to the cooking water. Thus, dilution of the composition of the cAQF by this factor results in a composition similar to composition data reported in the literature [6,8,17,22]. Furthermore, only the Frontier cultivar was assessed for aquafaba production, and thus differences observed may relate to the cultivar used to prepare AQF [13].

### 3.2. Functionality

The functionality of the four AQF samples was completed using the dry AQF or cAQF with adjustment in external water addition. The emulsion activity and stability were found to be significantly (*p* ≤ 0.05) higher for the cAQF compared to the AQF that had been dried. However, the drying operation had a small impact on the emulsion properties of the dried AQF since similar values were similar among the dried sample but less than the cAQF (Table 6). The results follow similar outcomes where fresh aquafaba generally outperformed AQF samples that were dried and rehydrated [18]. Differences in the emulsion properties observed in our AQF samples could be related to compositional difference not determined (e.g., saponins) in this study. For example, saponins are known emulsifiers and were thought to be one reason for the higher emulsion activity index of green lentils and chickpeas compared to other pulses [8,41]. There is evidence that saponins are degraded during heat processing [42,43], and thus the possibility exists that the added drying approaches may have caused reductions in saponins and thus lower emulsion activity and stability. Regardless of composition, the emulsion properties have been extensively demonstrated in mayonnaise applications [19,20,22,29].

The foaming capacity and stability were opposite of the emulsion properties where cAQF tended to have lower foaming capacity and stability compared with the samples that were dried (Table 6). The differences in the foaming properties might relate to the pH of the model system. In the model used for this experiment, the pH tended to be pH 6–7, which is not an optimal pH for AQF foaming [17]. Overall, cooking water or AQF can be a potential functional ingredient from pulses [44,45]. However, methods such as ultrasound processing [16] and high-pressure processing [46] can be used to improve foaming properties. For example, ultrasound processing improved the foaming capacity of chickpea cooking water by a factor of 2.1 [16].

### 3.3. Model Applications

#### 3.3.1. AQF Foam

**Figure 2:**
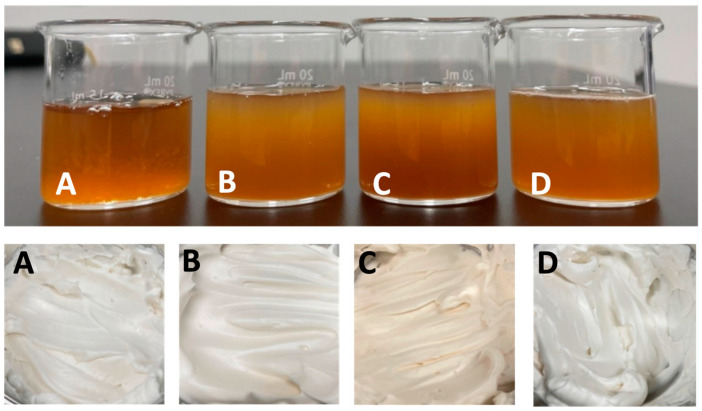
The appearance of the concentrated aquafaba (cAQF; (**A**)) and aquafaba samples prepared by rehydrating freeze- (**B**), tray- (**C**), or spray- (**D**) dried aquafaba to a 13% solids concentration (**upper images**) and their corresponding foams (**lower images**).

**Figure 3:**
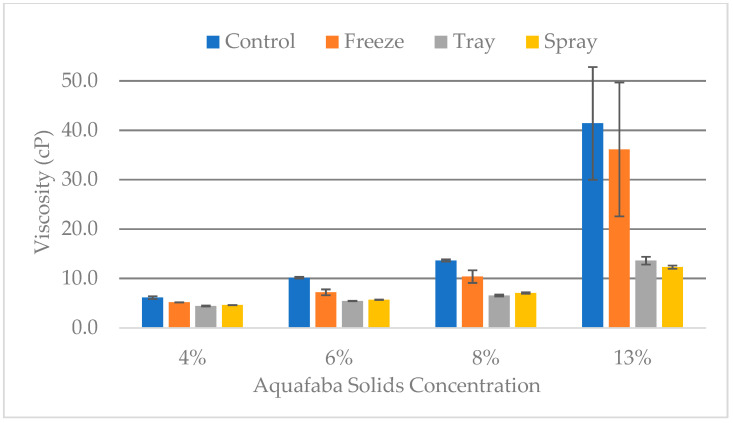
The viscosity (cP) of aquafaba samples prepared by rehydrating dry samples (freeze, spray, or tray) or diluting the reverse osmosis concentrated (cAQF or Control) sample to the various concentrations (4%, 6%, 8%, and 13%) of solids.

The AQF foam experiment was conducted to assess the impact of solids concentration (4%, 6%, 8%, and 13%) on the foaming properties of the AQF samples. Unlike the lab scale foaming experiment presented previously, this part of the experiment was completed to mimic the foaming done for meringue making at the kitchen scale. The AQF samples upon rehydration were visually brown and were not differentiated by color (Figure 2). As expected, the density increased with an increase in aquafaba concentration. For example, the densities of the solutions ranged from 0.99 g/cm^3^ in the 4% solids AQF to 1.024 g/cm^3^ in the 13% solids AQF. Statistically, only the 13% solids AQF was significantly (*p* ≤ 0.05) different from the other AQF concentrations. The higher concentration of protein and sugars in the 13% solids samples for example would be expected to higher density, which was supported by the density data. The greater density also impacted viscosity. The same trend was observed for the viscosity where the AQF with 13% solids was significantly (*p* ≤ 0.05) different from other AQF samples (Figure 3). However, the cAQF tended to be significantly more viscous than the other AQF samples except at the 13% solids concentration where the cAQF and AQF from the freeze-drying process were not significantly (*p* > 0.05) different (Figure 3).

Aquafaba produces a foam similar to the appearance of whipped egg whites. The foams formed stiff peaks and were light brown to bright white in color with a slight sheen (Figure 1). Unlike the dry AQF, significant differences in the lightness values were identified among foams prepared from the 13% solids AQF (Table 7). The foam prepared from the tray-dried sample tended to have the lowest lightness value (83.54) while the cAQF had the highest (86.97) lightness value. Furthermore, the foam prepared from tray-dried AQF had red (positive a value) and yellow (highest b value) that were significantly (*p* ≤ 0.05) higher than for the other AQF foams and had a brown appearance compared to the other foams. The L*, a*, and b* for the freeze- and spray-dried AQF showed that freeze-dried AQF was brighter and had a more negative a* value and a lower b* compared to spray-dried AQF. The color values followed the same trend as those for mayonnaise made from freeze- and spray-dried AQF [18].

In comparisons to the lab scale foaming capacity, the foam prepared at the kitchen scale showed substantially different results. In general, greater foaming capacity and stability were observed for the foams prepared by the kitchen scale approach. Furthermore, the lab scale method used AQF at a 1% solids concentration as a means to compare results to the literature reports on foaming of pulse flours and proteins, while the kitchen scale approach utilized 4% to 13% solids, and thus comparisons should not be made between the two different foaming tests. Furthermore, an acidulant (cream of tartar) was used in the preparation of the kitchen scale foams, which likely enhanced foaming of the AQF. Acidic pH or the addition of acid has been shown to improve the foaming capacity of AQF [14,23].

Regardless of concentration, the cAQF tended to have a foaming capacity of 1000%. However, this value was only significantly (*p* ≤ 0.05) different from the other AQF samples at the 4% solids concentration (Table 8). For concentrations between 6% and 13% solids, only the spray-dried AQF had lower foaming capacity than the other samples. Thus, no advantage in foaming capacity is gained when the solids concentrations are above 6% for most samples. It is possible that higher fiber or lower saponin contents in the spray-dried AQF was the cause of the lower foaming capacity [8,41].

Comparisons among processing methods generally showed that foam stability was not significantly (*p* > 0.05) different among samples except for spray-dried samples (Table 9). Overall, the very high foam stability in AQF in this study supports the reported high foaming stability for aquafaba obtained from commercially canned chickpea [6]. However, these authors did not report a concentration. Although statistical differences were found among AQF samples (Table 9), the values are probably not of practical differences. Thus, the foam observed from the AQF with 6% concentration of solids provides evidence for the proper usage level since no benefit was gained at higher concentrations (8% and 13% solids). Emulsion capacity and stability are similar to foaming capacity and stability in that they rely on protein functionality. He et al. [13] found that emulsion capacity and stability were dependent on solids concentration, with higher solids concentrations having better emulsion properties. They evaluated aquafaba samples with solid content between 5.8% and 7.6%. Our data show similar trends in that the low concentration of solids (4%) also had lower foam capacities and stabilities, but above 6% no additional benefit was gained.

#### 3.3.2. Cakes

Based on foaming results, 6% aquafaba was identified as a sufficient replacement for traditional eggs in a cake formula. Additionally, cakes were produced in two ways: first, the aquafaba was foamed and then added to the cake, and second, liquid aquafaba was added directly into the formulation. It was determined the foaming step was not necessary to produce desirable results; thus, the foaming step prior to mixing with the cake mix was eliminated.

The cake moistures (cAQF (23.6%), spray (23.7%), egg (24.6%), tray (24.7%), and freeze (25.3%)) were not significantly (*p* > 0.05) different. In contrast, water activities (cAQF (0.78), spray (0.81), tray (0.82), freeze (0.83), and egg (0.86)) were significant among the cakes. The water activity of the cake made with cAQF was significantly (*p* ≤ 0.05) lower compared to all cakes except the cakes made with spray-dried AQF. For the cake made with egg, the water activity was significantly (*p* ≤ 0.05) higher than water activities of other cakes except cake made with the freeze-dried AQF. The cakes prepared in this study tended to have lower moisture content and water activities compared to a previous report [27] but slightly higher moisture content compared to a sponge cake model [7]. Although not the same systems, lower moisture content was observed in gluten-free bread fortified with AQF [28].

**Figure 4:**
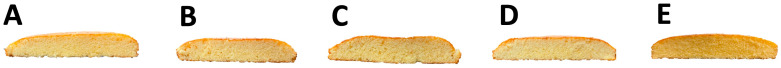
Cake cross-section images of vanilla cakes made with aquafaba ((**A**) = cAQF, (**B**) = freeze-dried AQF, (**C**) = tray-dried AQF, and (**D**) = spray-dried AQF) or without ((**E**) = egg).

Only the cakes made with egg and tray-dried AQF had significantly different C-cell brightness values. Other cakes had brightness values that were not significantly different. The yellow color of the cake mix was sufficient to mask the color differences among the AQF samples. The volume, symmetry, and uniformity index were not significantly (*p* > 0.05) different for the cakes, although the cake with tray-dried AQF had a slight indent compared to other cakes (Figure 4). Others [7,27] have found similar or slightly higher values for these cake properties. However, the differences in formulations likely contributed to differences in cake properties. Similar observations regarding increased specific volumes were reported for gluten free bread [28].

All of the textural data were significantly (*p* ≤ 0.05) higher for the cakes made with egg compared to the AQF (Table 10). The only exception was springiness and cohesiveness, where these values for the cakes made with freeze-dried AQF and cAQF were not significantly different from the cake made with eggs. None of the cakes made with AQF had texture values that were significant. Cake structure is heavily dependent on the interactions between egg proteins or between egg protein and wheat protein and starch gelatinization during the baking process [24,25,26]. Chewiness, springiness, and cohesiveness were also reported to be higher for a sponge cake made with egg white compared to AQF [7]. Increasing concentration (25–35%) of AQF in cake formulation resulted in lower firmness [27], thus supporting the observation that AQF likely inhibits starch retrogradation that is responsible for firmness in products like cakes. Cakes tend to have less springiness if insufficient cell wall structure is developed between proteins and starch during baking [24,25,26]. Thus, differences observed in cakes made with AQF compared to eggs likely lacked sufficient cell wall structure. This potential mechanism is further supported by the lower hardness values reported for gluten-free bread made with AQF from chickpeas [28,47] and lima beans [9]. In contrast to the data obtained by the texture analyzer, sensory scores varied among samples.

In the texture analysis, firmness was approximately 1.4–1.5 times higher for the cakes made with eggs (Table 10). The firmness of cakes has been attributed to the gel-forming capacity of starch [26]. The starch component was expected to be similar for all cakes since a standard commercial cake mix was used for all cakes. Thus, the lower firmness of AQF-containing cakes compared to the cakes made with egg was potentially due to the interference of starch gelatinization by the AQF components and the weak interaction between the AQF protein and starch. However, this difference in texture did not impact sensory panelist rating of the samples, as only the cake made with tray-dried AQF had significantly (*p* ≤ 0.05) lower texture acceptability ratings (Table 11). The flavor and overall acceptability followed the same trend of lower acceptability scores for the cake made with tray-dried AQF. Furthermore, the cakes made with freeze- and tray-dried AQF had significantly (*p* ≤ 0.05) lower ratings for appearance compared to the other cakes. The cake made with egg was denser than the cakes made with the freeze- and tray-dried AQF, which was noted by multiple panelists and was likely the reason for the higher acceptability score for the cake made with egg. Similar non-significant sensory outcomes comparing cake with and without AQF were also reported for pound cake [27].

#### 3.3.3. Cookies

**Figure 5:**
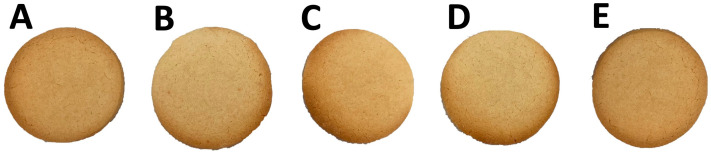
Cookies made with aquafaba ((**A**) = cAQF, (**B**) = freeze-dried AQF, (**C**) = tray-dried AQF, and (**D**) = spray-dried AQF) or without ((**E**) = egg).

The cookies (Figure 5) had similar appearance, physical characteristics, and sensory characteristics. However, some differences were observed among the cookies. In contrast to cakes, no significant differences in water activities (0.40–0.43) were observed among cookies. The moisture content (%) of the cookies containing cAQF tended to be significantly (*p* ≤ 0.05) higher than the other cookies through day 8 of storage (Table 12). However, by day 14, moisture content was not significantly different among cookies made with AQF. With few exceptions, the cookies containing egg had significantly lower moisture content (Table 12).

Physical parameters of the cookies at day one show that the cookie diameter was not impacted by the presence of AQF (Table 13) while cookie thickness was generally lower for cookies made with AQF as opposed to egg. The spread factor for cookies made with eggs tended to be less than cookies made with AQF. However, only the cookies made with cAQF and tray-dried AQF had significantly (*p* ≤ 0.05) higher spread factors than cookies made with eggs. Only the cookies prepared with the cAQF had significantly (*p* ≤ 0.05) lower hardness compared to the egg-based cookies (Table 13). Comparisons of hardness values of cookies made with AQF shows that only the cookies made with tray-dried AQF had significantly different hardness values than cookies made with cAQF. The fracturability is the distance traveled by the texture analyzer probe before the cookie fractures or reaches a peak force. This indicates that the cookie is more flexible or compressible as the distance increases. The general relationship is that as hardness increases, fracturability generally decreases. However, this relationship did not follow as expected. Instead, the higher the hardness value for the cookie, the greater was the fracturability (Table 13). Significant (*p* ≤ 0.05) differences were found for fracturability, where cookies made with egg had higher fracturability values while cookies made with cAQF had lower values. The observed texture results indicate that the significantly higher moisture content in cAQF cookies likely contributed to the lower texture values. Although moisture content increased during the 14-day storage, the hardness value for cookies with cAQF increased and was no longer significantly (*p* > 0.05) different from hardness values of other cookies (Table 14). No significant differences in fracturability between cookies made with AQF were observed at day 14; however, cookies made with spray- or tray-dried AQF had significantly (*p* ≤ 0.05) lower fracturability values compared to the cookies made with egg (Table 15). There was no clear trend for changes in fracturability over the 14-day storage. In some samples no change occurred from the day 1 storage while in other cases an increase or decrease was observed. There is evidence that starch is an important component that imparts texture; however, the molecular structure of starch did not influence cookie hardness and fracturability [48]. These authors [48] suggested that the continuous glassy sucrose–water matrix embedded with ungelatinized starch granules contributed to the textural features of sugar-snap cookies. Thus, it is possible that the AQF disrupted the matrix and caused the AQF containing cookies to have less hardness and fracturability.

With few exceptions, the cookie L*, a*, and b* values in general were not significant among formulation. Mean differences in the a* values of cookies made with spray-dried AQF (9.01) and cAQF (3.03) or eggs (3.90) were significant (*p* ≤ 0.05) while other mean comparisons were not significant. Cookies made with cAQF had a significantly (*p* ≤ 0.05) lower b* value (27.63) compared to cookies made with spray-dried AQF (29.63) or eggs (30.11). No other b* mean comparisons were significant for the cookies. In general, the lack of differences in cookie L*, a*, and b* values are supported by the sensory data where cookie appearance was not significantly (*p* > 0.05) different among cookies prepared from the different formulations (Table 16). Flavor, texture, and overall sensory scores were significantly (*p* ≤ 0.05) greater for the cookies made with either cAQF or spray-dried AQF compared to the cookies made with egg. The general trend of higher sensory scores compared to the egg-based cookie also applies to other AQF samples, but in some cases the scores were not statistically different.

## 4. Conclusions

The hypothesis of this research was that concentration and drying methods will impact the quality or functionality of chickpea AQF in model foam and emulsion systems or impact the quality of cakes and cookies. The research supports the idea that the concentration of AQF solids did impact some functional properties. However, in the application of AQF in cakes, the concentration of 6% was ideal for producing cakes with similar or better properties than cake made with eggs. Thus, a recommendation to limit the solids concentration to 6% in cake applications is a recommendation. Overall, the drying process followed by rehydration was a convenient way to handle AQF. Furthermore, the resulting sensory and quality of products made with cAQF or dried AQF was similar or better than the products made with egg. Thus, the data support the idea that dried AQF was an effective egg replacer in bakery products. However, more in-depth assessment should be completed to determine the specific components responsible for the functionality. Furthermore, changes (composition, structural changes in protein, etc.) that occurred as a result of drying processes should be investigated if AQF is to become an egg replacer.

## Figures and Tables

**Table 1:** Formulations of the cakes made with Jiffy^®^ golden yellow cake mix, with or without egg and aquafaba.

Ingredient	Egg Formula (%)	Dried Aquafaba Formula (%)	Reverse Osmosis Aquafaba Formula (%) ^1^
Cake Mix	247.5 g (60.7)	255 g (62.6)	255 g (62.6)
Water	115 g (28.2)	143.5 g (35.2)	79.7 g (19.6)
Egg	45.25 (11.1)	0	0
Aquafaba	0	9.2 g (2.2)	73.0 g (17.9)

^1^ The cAQF contained significantly more water than the dried sample and thus differences between formulas reflect a water weight difference.

**Table 2:** Formulations (g and %) of the cookies made with or without egg and aquafaba.

Ingredient	Egg Formula (%)	Dried Aquafaba Formula (%) ^1^	Concentrated Aquafaba Formula (%) ^2^
Flour	209.5 g (41.9)	209.5 g (41.9)	209.5 g (41.9)
Butter	131 g (26.2)	131 g (26.2)	131 g (26.2)
Sugar	125.5 g (25.1)	125.5 g (25.1)	125.5 g (25.1)
Egg	28.5 g (5.7)	0	0
Aquafaba	0	7.41 g (1.5)	28.5 g (5.7)
Water	0	21.09 g (4.2)	0
Vanilla	3.0 g (0.6)	3.0 g (0.6)	3.0 g (0.6)
Baking Soda	1.5 g (0.3)	1.5 g (0.3)	1.5 g (0.3)
Baking Powder	1.0 g (0.2)	1.0 g (0.2)	1.0 g (0.2)

^1^ The dried aquafaba included spray-, freeze-, or tray-dried aquafaba combined with water to balance the water weight associated with that of egg. ^2^ The cAQF (reverse osmosis aquafaba) contained significantly more water than the dried sample and thus differences between formulas reflect a water weight difference.

**Table 3:** The L*, a*, and b* values ^1^ of the dried AQF samples.

Sample	L*	a*	b*
Spray-Dried	86.39 ± 0.08 ^A^	−0.31 ± 0.01 ^C^	14.68 ± 0.56 ^B^
Freeze-Dried	82.69 ± 0.02 ^A^	0.71 ± 0.01 ^B^	15.54 ± 0.42 ^B^
Tray-Dried	65.52 ± 21.20 ^A^	1.70 ± 0.03 ^A^	23.10 ± 0.19 ^A^

^1^ Values are reported as mean ± standard deviation. Values (n = 4) with different letters in the same column are significantly different at *p* ≤ 0.05.

**Table 4:** Proximate analysis of the aquafaba obtained by reverse osmosis (cAQF) or drying by freeze, tray, or spray drying.

Sample	Starch (%) ^1^	Protein (%)	Lipid (%)	Ash (%)	TDF (%) ^3^
Freeze	0.3 ± 0.2	21.7 ± 1.0	0.1 ± 0.01	13.2 ± 0.4	6.0 ± 1.5
Spray	0.3 ± 0.2	22.6 ± 0.2	0.0 ± 0.00	13.1 ± 0.4	8.7 ± 0.4
Tray	0.3 ± 0.2	21.9 ± 0.9	0.0 ± 0.00	13.4 ± 0.3	7.0 ± 1.0
cAQF ^2^	0.041 ± 0.02	3.0 ± 0.01	0.0 ± 0.00	1.8 ± 0.14	0.8 ± 0.25

^1^ Mean (dry weight basis) followed by standard deviation (n = 2). ^2^ Estimated composition (as is basis) based on data from freeze-dried sample. ^3^ Total dietary fiber.

**Table 5:** Sugar analysis of the aquafaba obtained by reverse osmosis (cAQF) or drying by freeze, tray, or spray drying.

Sample	Glucose (%) ^1^	Sucrose (%)	Fructose (%)	Stachyose (%)	Raffinose (%)
Freeze	0.3 ± 0.1	14.2 ± 0.2	0.2 ± 0.0	10.0 ± 0.1	4.0 ± 0.0
Spray	0.3 ± 0.1	14.2 ± 0.1	0.2 ± 0.0	10.3 ± 0.4	4.0 ± 0.2
Tray	0.1 ± 0.1	12.7 ± 0.5	0.1 ± 0.1	9.7 ± 0.1	3.9 ± 0.1
cAQF ^2^	0.01 ± 0.01	2.0 ± 0.12	0.00 ± 0.00	1.4 ± 0.09	0.5 ± 0.03

^1^ Mean (dry weight basis) followed by standard deviation (n = 2). ^2^ Estimated composition (as is basis) based on data from freeze-dried sample.

**Table 6:** Emulsion and foaming properties of aquafaba.

Aquafaba	Emulsion Activity (%) ^1^	Emulsion Stability (%)	Foaming Capacity (%)	Foaming Stability (%)
cAQF	54.1 ± 1.2 ^A^	55.8 ± 1.7 ^A^	181.0 ± 40.4 ^B^	33.4 ± 11.1 ^B^
Spray-Dried	42.0 ± 1.6 ^B^	43.8 ± 7.3 ^B^	205.0 ± 12.6 ^A,B^	74.8 ± 6.7 ^A^
Freeze-Dried	41.3 ± 1.3 ^B^	44.1 ± 4.1 ^B^	201.7 ± 12.6 ^B^	74.7 ± 7.6 ^A^
Tray-Dried	40.5 ± 2.4 ^B^	44.1 ± 4.9 ^B^	231.7 ± 18.4 ^A^	70.3 ± 3.5 ^A^

^1^ Values are reported as mean ± standard deviation. Values (n = 4) with different letters in the same column are significantly different at *p* ≤ 0.05.

**Table 7:** The L*, a*, and b* color value ^1^ for foam produced from the 13% solids aquafaba.

Foam	L*	a*	b*
cAQF	86.97 ± 0.10 ^A^	−0.45 ± 0.01 ^D^	9.56 ± 0.11 ^D^
Freeze-Dried	85.83 ± 0.18 ^B^	−0.37 ± 0.01 ^C^	10.71 ± 0.18 ^C^
Spray-Dried	84.89 ± 0.02 ^C^	−0.26 ± 0.02 ^B^	11.94 ± 0.13 ^B^
Tray-Dried	83.54 ± 0.16 ^D^	0.14 ± 0.01 ^A^	14.25 ± 0.11 ^A^

^1^ Values are reported as mean ± standard deviation. Values (n = 4) with different letters in the same column are significantly different at *p* ≤ 0.05.

**Table 8:** Foam capacity (%) ^1^ of aquafaba at solids concentration of 4% to 13%.

Aquafaba	4%	6%	8%	13%
cAQF	1000 ± 0 ^A,a^	1000 ± 0 ^A,a^	1000 ± 0 ^A,a^	1000 ± 0 ^A,a^
Spray-Dried	790 ± 30 ^C,b^	898 ± 42 ^B,a^	950 ± 11 ^B,a^	743 ± 95 ^B,b^
Freeze-Dried	973 ± 12 ^B,a^	983 ± 13 ^A,a^	1000 ± 0 ^A,a^	1000 ± 0 ^A,a^
Tray-Dried	963 ± 12 ^B,b^	1000 ± 0 ^A,a^	1000 ± 0 ^A,a^	1000 ± 0 ^A,a^

^1^ Values are reported as mean ± standard deviation. Values (n = 12) with different uppercase letters in the same column are significantly different at *p* ≤ 0.05. Values (n = 12) with different lowercase letters in the same row are significantly different at *p* ≤ 0.05.

**Table 9:** Foam stability (%) ^1^ of aquafaba at solids concentration of 4% to 13%.

Aquafaba	4% ^1^	6%	8%	13%
cAQF	99.8 ± 0.1 ^A,a^	99.9 ± 0.1 ^A,a^	100 ± 0 ^A,a^	100 ± 0 ^A,a^
Spray-Dried	99.2 ± 0.3 ^B,b^	99.9 ± 0.2 ^A,a^	100 ± 0 ^A,a^	100 ± 0 ^A,a^
Freeze-Dried	99.8 ± 0.1 ^A,b^	100 ± 0 ^A,a^	100 ± 0 ^A,a^	99.6 ± 0.1 ^B,b^
Tray-Dried	99.9 ± 0.1 ^A,b^	100 ± 0 ^A,a^	100 ± 0 ^A,a^	100 ± 0 ^A,a^

^1^ Values are reported as mean ± standard deviation. Values (n = 12) with different uppercase letters in the same column are significantly different at *p* ≤ 0.05. Values (n = 12) with different lowercase letters in the same row are significantly different at *p* ≤ 0.05.

**Table 10:** Texture properties of cakes made with egg or aquafaba (6% solids concentration).

Formula	Firmness (g) ^1^	Chewiness (g)	Gumminess (g)	Springiness	Cohesiveness	Resilience
Egg	415 ± 18 ^A^	425 ± 12 ^A^	285 ± 5 ^A^	1.51 ± 0.02 ^A^	0.69 ± 0.02 ^A^	0.41 ± 0.01 ^A^
cAQF	290 ± 22 ^B^	183 ± 27 ^B^	183 ± 19 ^B^	1.00 ± 0.06 ^B^	0.63 ± 0.02 ^A,B^	0.34 ± 0.02 ^B^
Spray-Dried	266 ± 2 ^B^	153 ± 3 ^B^	157 ± 3 ^B^	0.97 ± 0.02 ^B^	0.59 ± 0.01 ^B^	0.32 ± 0.01 ^B^
Freeze-Dried	283 ± 4 ^B^	231 ± 51 ^B^	178 ± 10 ^B^	1.29 ± 0.22 ^A, B^	0.63 ± 0.2 ^A,B^	0.34 ± 0.02 ^B^
Tray-Dried	275 ± 3 ^B^	152 ± 6 ^B^	163 ± 3 ^B^	0.93 ± 0.02 ^B^	0.59 ± 0.01 ^B^	0.32 ± 0.01 ^B^

^1^ Values are reported as mean ± standard deviation. Values (n = 6) with different uppercase letters in the same column are significantly different at *p* ≤ 0.05.

**Table 11:** Sensory acceptability ratings for cakes made with egg or aquafaba (6% solids concentration).

Formula	Appearance ^1^	Flavor	Texture	Overall
Egg	7.7 ± 1.0 ^A^	6.9 ± 1.6 ^A^	6.9 ± 1.6 ^A^	7.1 ± 1.3 ^A^
cAQF	7.5 ± 1.2 ^A,B,C^	7.0 ± 1.4 ^A^	6.9 ± 1.6 ^A^	7.0 ± 1.4 ^A^
Spray-Dried	7.6 ± 1.1 ^A,B^	7.1 ± 1.4 ^A^	6.9 ± 1.6 ^A^	7.1 ± 1.3 ^A^
Freeze-Dried	7.2 ± 1.3 ^B,C^	7.0 ± 1.5 ^A^	6.70 ± 1.6 ^A,B^	7.0 ± 1.5 ^A^
Tray-Dried	7.0 ± 1.5 ^C^	5.9 ± 2.1 ^B^	6.2 ± 1.8 ^B^	6.1 ± 1.8 ^B^

^1^ Values are reported as mean ± standard deviation. Values with different uppercase letters in the same column are significantly different at *p* ≤ 0.05.

**Table 12:** Moisture percentage in cookies made with or without aquafaba and stored for up to 14 days.

Formula	Day 1 ^1^	Day 4	Day 8	Day 14
Egg	2.75 ± 0.35 ^B,a,b^	2.68 ± 0.48 ^B,b^	3.17 ± 0.38 ^B,a,c^	3.50 ± 0.46 ^C,c^
cAQF	3.56 ± 0.34 ^A, a^	4.17 ± 0.38 ^A,b^	3.64 ± 0.27 ^A,a^	4.30 ± 0.42 ^A,B,b^
Spray-Dried	2.30 ± 0.37 ^C,a^	2.37 ± 0.29 ^C,a^	2.78 ± 0.22 ^C,b^	4.03 ± 0.41 ^B,C,c^
Freeze-Dried	2.84 ± 0.27 ^B,a^	2.94 ± 0.36 ^B,a^	3.26 ± 0.12 ^B,b^	4.19 ± 0.32 ^A,B,c^
Tray-Dried	2.67 ± 0.40 ^B,C,a^	2.90 ± 0.25 ^B,a,b^	3.35 ± 0.20 ^B,b^	4.61 ± 0.70 ^A,c^

^1^ Values are reported as mean ± standard deviation. Values (n = 12) with different uppercase letters in the same column are significantly different at *p* ≤ 0.05. Values (n = 12) with different lowercase letters in the same row are significantly different at *p* ≤ 0.05.

**Table 13:** Physical parameters of cookies made with or without aquafaba and measure 24 h after preparation.

Formulation	Diameter (mm) ^1^	Thickness (mm)	Spread Factor (D/T)	Hardness (g)	Fracturability (mm)
Egg	67.3 ± 0.17 ^A^	8.2 ± 0.31 ^A^	8.2 ± 0.24 ^B^	1808 ± 388 ^A^	45.35 ± 0.52 ^A^
cAQF	65.1 ± 0.69 ^A^	7.0 ± 0.06 ^B^	9.4 ± 0.02 ^A^	1274 ± 323 ^B^	42.91 ± 0.45 ^C^
Spray-Dried	67.3 ± 0.92 ^A^	7.5 ± 0.37 ^B^	9.0 ± 0.32 ^A,B^	1621 ± 157 ^A,B^	44.28 ± 0.54 ^B^
Freeze-Dried	65.7 ± 0.23 ^A^	7.6 ± 0.18 ^A,B^	8.7 ± 0.24 ^A,B^	1687 ± 285 ^A,B^	44.45 ± 0.27 ^B^
Tray-Dried	66.5 ± 1.09 ^A^	7.2 ± 0.12 ^B^	9.3 ± 0.01 ^A^	1834 ± 101 ^A^	44.52 ± 0.36 ^B^

^1^ Values are reported as mean ± standard deviation. Values (n = 12) with different uppercase letters in the same column are significantly different at *p* ≤ 0.05.

**Table 14:** Hardness (g) of cookies made with or without aquafaba and stored for up to 14 days.

Ingredient	Day 1 ^1^	Day 4	Day 8	Day 14
Egg	1808 ± 388 ^A^	1876 ± 253 ^A^	1705 ± 233 ^A,B^	1779 ± 321 ^A^
cAQF	1274 ± 323 ^B^	1350 ± 325 ^B^	1224 ± 227 ^B^	1603 ± 403 ^A^
Spray-Dried	1621 ± 157 ^A,B^	1790 ± 172 ^A^	1320 ± 533 ^A,B^	1718 ± 82 ^A^
Freeze-Dried	1687 ± 285 ^A,B^	1796 ± 185 ^A^	1774 ± 220 ^A^	1967 ± 215 ^A^
Tray-Dried	1834 ± 101 ^A^	1700 ± 202 ^A^	1638 ± 109 ^A,B^	1630 ± 149 ^A^

^1^ Values are reported as mean ± standard deviation. Values (n = 12) with different uppercase letters in the same column are significantly different at *p* ≤ 0.05.

**Table 15:** Fracturability (mm) in cookies made with or without aquafaba and stored for up to 14 days.

Ingredient	Day 1 ^1^	Day 4	Day 8	Day 14
Egg	45.35 ± 0.52 ^A,a^	45.37 ± 0.32 ^A,a^	45.05 ± 0.31 ^A,a^	45.16 ± 0.41 ^A,a^
cAQF	42.91 ± 0.45 ^C, b^	43.64 ± 0.34 ^C,a,b^	42.88 ± 0.55 ^C,b^	44.18 ± 0.93 ^A,B,a^
Spray-Dried	44.28 ± 0.54 ^B,a^	43.99 ± 0.51 ^B,C,a^	43.40 ± 1.28 ^B,C,a^	44.00 ± 0.57 ^B,a^
Freeze-Dried	44.45 ± 0.27 ^B,a^	44.48 ± 0.29 ^B,a^	44.42 ± 0.37 ^A,B,a^	44.36 ± 0.33 ^A,B,a^
Tray-Dried	44.52 ± 0.36 ^B,a^	43.74 ± 0.58 ^C,b^	43.77 ± 0.43 ^B,C,b^	43.53 ± 0.14 ^B,b^

^1^ Values are reported as mean ± standard deviation. Values (n = 12) with different uppercase letters in the same column are significantly different at *p* ≤ 0.05. Values (n = 12) with different lowercase letters in the same row are significantly different at *p* ≤ 0.05.

**Table 16:** Sensory acceptability ratings for cookies made with egg or aquafaba.

Formula	Appearance ^1^	Flavor	Texture	Overall
Egg	7.0 ± 1.3 ^A^	6.3 ± 1.7 ^B^	6.9 ± 1.5 ^B^	6.6 ± 1.6 ^C^
cAQF	7.4 ± 1.1 ^A^	7.3 ± 1.4 ^A^	7.5 ± 1.2 ^A^	7.4 ± 1.2 ^A^
Spray-Dried	7.4 ± 1.2 ^A^	7.3 ± 1.2 ^A^	7.4 ± 1.3 ^A^	7.5 ± 1.1 ^A^
Freeze-Dried	7.2 ± 1.2 ^A^	7.2 ± 1.5 ^A^	7.2 ± 1.4 ^A,B^	7.2 ± 1.5 ^A,B^
Tray-Dried	7.3 ± 1.2 ^A^	6.8 ± 1.6 ^A,B^	7.2 ± 1.3 ^A,B^	6.9 ± 1.8 ^B,C^

^1^ Values are reported as mean ± standard deviation. Values with different uppercase letters in the same column are significantly different at *p* ≤ 0.05.

## Data Availability

Data is contained within the article.
